# Mono and Multiple Tumor-Targeting Ligand-Coated Ultrasmall Gadolinium Oxide Nanoparticles: Enhanced Tumor Imaging and Blood Circulation

**DOI:** 10.3390/pharmaceutics14071458

**Published:** 2022-07-12

**Authors:** Son Long Ho, Huan Yue, Sangyeol Lee, Tirusew Tegafaw, Mohammad Yaseen Ahmad, Shuwen Liu, Abdullah Khamis Ali Al Saidi, Dejun Zhao, Ying Liu, Sung-Wook Nam, Kwon Seok Chae, Yongmin Chang, Gang Ho Lee

**Affiliations:** 1 Department of Chemistry, College of Natural Sciences, Kyungpook National University, Taegu 41566, Korea; sonlongh@gmail.com (S.L.H.); yuehuan888@gmail.com (H.Y.); tirukorea@gmail.com (T.T.); yaseen.knu@gmail.com (M.Y.A.); liushuwen0701@gmail.com (S.L.); abdullah_al_saidi@hotmail.com (A.K.A.A.S.); djzhao.chem@gmail.com (D.Z.); ly1124161@gmail.com (Y.L.); 2 Department of Medical & Biological Engineering, Kyungpook National University, Taegu 41944, Korea; tkdduf1405@knu.ac.kr; 3 Department of Molecular Medicine, School of Medicine, Kyungpook National University, Taegu 41944, Korea; nams@knu.ac.kr; 4 Department of Biology Education, Teachers’ College, Kyungpook National University, Taegu 41566, Korea; kschae@knu.ac.kr

**Keywords:** ultrasmall Gd_2_O_3_ nanoparticle, folic acid, cRGD, multiple tumor-targeting ligand, tumor imaging, blood circulation enhancement

## Abstract

Hydrophilic and biocompatible PAA-coated ultrasmall Gd_2_O_3_ nanoparticles (d_avg_ = 1.7 nm) were synthesized and conjugated with tumor-targeting ligands, i.e., cyclic arginylglycylaspartic acid (cRGD) and/or folic acid (FA). FA-PAA-Gd_2_O_3_ and cRGD/FA-PAA-Gd_2_O_3_ nanoparticles were successfully applied in U87MG tumor-bearing mice for tumor imaging using T_1_ magnetic resonance imaging (MRI). cRGD/FA-PAA-Gd_2_O_3_ nanoparticles with multiple tumor-targeting ligands exhibited higher contrasts at the tumor site than FA-PAA-Gd_2_O_3_ nanoparticles with mono tumor-targeting ligands. In addition, the cRGD/FA-PAA-Gd_2_O_3_ nanoparticles exhibited higher contrasts in all organs, especially the aorta, compared with those of the FA-PAA-Gd_2_O_3_ nanoparticles, because of the blood cell hitchhiking effect of cRGD in the cRGD/FA-PAA-Gd_2_O_3_ nanoparticles, which prolonged their circulation in the blood.

## 1. Introduction

Molecular imaging is an important tool for tumor diagnosis [1,2,3]. Currently available imaging modalities include magnetic resonance imaging (MRI) (25–100 μm, 10^−11^–10^−12^ M), X-ray computed tomography (50–200 μm, not well characterized), fluorescent imaging (2–3 mm, 10^−9^–10^−12^ M), ultrasound imaging (50–200 μm, not well characterized), positron emission tomography (1–2 mm, 10^−11^–10^−12^ M), and single-photon emission computed tomography (1–2 mm, 10^−11^–10^−12^ M); the numbers in parentheses are spatial resolution and sensitivity in terms of imaging probe concentration, respectively [3]. Among these, MRI is a non-invasive imaging technique commonly used for disease diagnosis owing to its high spatial resolution with no depth limit [2,3]. Currently, various Gd-chelates are commercially available as MRI contrast agents. In addition, recent studies have demonstrated that Gd-based nanoparticles can provide better contrast than molecular Gd-chelates [4,5]. Nanoparticles facilitate enhanced tumor imaging compared with small molecules owing to their advanced imaging properties and facile conjugation with tumor-targeting ligands [4,5]. Furthermore, nanoparticles allow diverse theranostic applications via surface functionalization, leading to improved tumor treatments [5,6]. Moreover, the number of nanoparticles delivered to the tumor can be enhanced using multiple tumor-targeting ligands, as this strategy can overcome the receptor saturation phenomenon observed in mono tumor-targeting ligand-coated nanoparticles [7,8,9].

Conventional large nanoparticles adhere to blood plasma proteins, including albumins and serum (termed as opsonization), during circulation [10,11]. As a result, they can be taken up by the reticuloendothelial system (RES), which consists of phagocytic cells [12,13,14,15]. This limits the lifetime and availability of nanoparticles in the blood and reduces their therapeutic efficacy. However, opsonization and RES uptake can be reduced by coating nanoparticles with hydrophilic ligands, such as polyethylene glycols (PEGs) [6], and restricting their hydrodynamic diameters to 10–100 nm [14,15]. Additionally, this size range facilitates their delayed renal excretion, as it is larger than the kidney glomerular epithelial filtration slit (~4 nm) [16]. Consequently, tumor imaging and therapy can be improved by prolonged blood circulation of such nanoparticles.

Among others, ultrasmall gadolinium oxide (Gd_2_O_3_) nanoparticles are of special interest, as Gd possesses unique and excellent theranostic properties [17]. The pure high-spin magnetic moment of Gd (S = 7/2) makes it an ideal core metal ion for commercial application as a T_1_ MRI contrast agent [18,19,20]. In addition, Gd has the highest neutron capture cross-section (σ = 254,000 barns) among stable radioisotopes [21,22,23]. These properties render Gd-based chemicals extremely useful as MRI-guided neutron capture therapeutic agents for malignant tumors [24]. Notably, ultrasmall Gd_2_O_3_ nanoparticles have considerably higher longitudinal water proton spin relaxivities (r_1_) than those of commercially available Gd-chelates [25,26,27,28,29,30]. Moreover, their r_2_/r_1_ ratios (r_2_ = transverse water proton spin relaxivity) are close to one; therefore, they can act as high-performance T_1_ MRI contrast agents.

In the present study, ultrasmall Gd_2_O_3_ nanoparticles were synthesized using a one-pot polyol method and grafted with polyacrylic acid (PAA; M_w_ = ~1800 amu) to form water-soluble and biocompatible nanoparticle colloids in aqueous media. PAA-Gd_2_O_3_ nanoparticles were further conjugated with folic acid (FA) and/or cyclic arginylglycylaspartic acid (cRGD) tumor-targeting ligands to prepare FA-PAA-Gd_2_O_3_ and cRGD/FA-PAA-Gd_2_O_3_ nanoparticles, respectively. FA targets the overexpressed folate receptors on tumor cells and facilitates nanoparticle penetration [31,32,33], whereas cRGD peptides target the overexpressed integrin receptors (e.g., α_v_β_3_) on tumor cells, which are associated with tumor angiogenesis and metastasis [34,35,36,37,38,39]. Therefore, FA and cRGD have been extensively used as tumor targeting ligands for tumor imaging and diagnosis [31,34,37,38,39] as well as drug delivery during chemotherapy, including drugs such as of doxorubicin and paclitaxel [32,33,35,36,38] in vitro and in vivo. Drugs can be delivered to tumor cells after conjugation with magnetic nanoparticles [32], liposomes [33], commercial imaging agents [34], micelles [35], bacteria [36], gold nanoparticles [37], polymer nanoparticles [38], and quantum dots [39]. In this study, T_1_ MR images of the tumor were used to investigate the enhanced tumor imaging of cRGD/FA-PAA-Gd_2_O_3_ nanoparticles compared with FA-PAA-Gd_2_O_3_ nanoparticles. In addition, T_1_ MR images of the aorta, kidneys, and liver were used to investigate the effect of cRGD in cRGD/FA-PAA-Gd_2_O_3_ nanoparticles on blood circulations as compared with FA-PAA-Gd_2_O_3_ nanoparticles.

## 2. Materials and Methods

### 2.1. Materials

Gadolinium(III) chloride hexahydrate (99.9%), sodium hydroxide (>99.9%), triethylene glycol (TEG) (99%), PAA (M_w_ = ~1800 amu), dimethyl sulfoxide (DMSO) (99.9%), N,N′-dicyclohexylcarbodiimide (DCC) (99%), 4-(dimethylamino) pyridine (DMAP) (>9%), tert-butyl N-(2-aminoethyl) carbamate (EDA-Boc) (>98%), triethylamine (TEA) (>99%), trifluoroacetic acid (TFA) (99%), N-hydroxysuccinimide (NHS) (98%), 1-ethyl-3 (3-dimethylaminopropyl) carbodiimide hydrochloride (EDC∙HCl) (97%), FA (>97%), Roswell Park Memorial Institute (RPMI)-1640, Dulbecco’s Modified Essential Medium (DMEM), sterile phosphate-buffered saline (PBS) solution, and dialysis tube (molecular weight cut-off (MWCO) = 1000 and 2000 amu) were procured from Sigma-Aldrich (St. Louis, MO, USA). cRGD (cRGDyk: Arg-Gly-Asp-D-Tyr-Lys) was procured from Vivitide (Gardner, MA, USA). Ethyl acetate (99.9%), chloroform (99.9%), and ethanol (99.99%) were purchased from Duksan (Ansan, Korea). All reagents and materials were used as received. Nanoparticles were initially washed with ethanol, then finally washed with triple-distilled water to prepare nanoparticle suspensions.

### 2.2. Preparation of FA-PAA-Gd_2_O_3_ Nanoparticles

**Figure 1:**
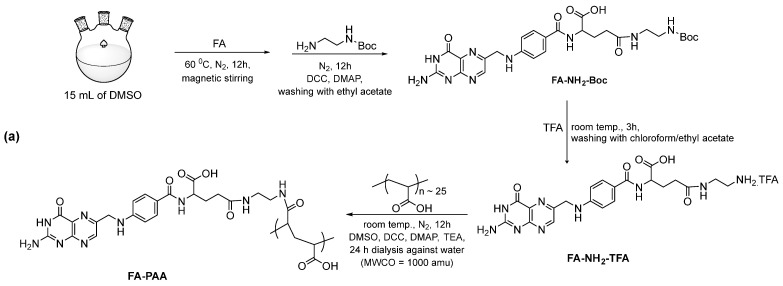

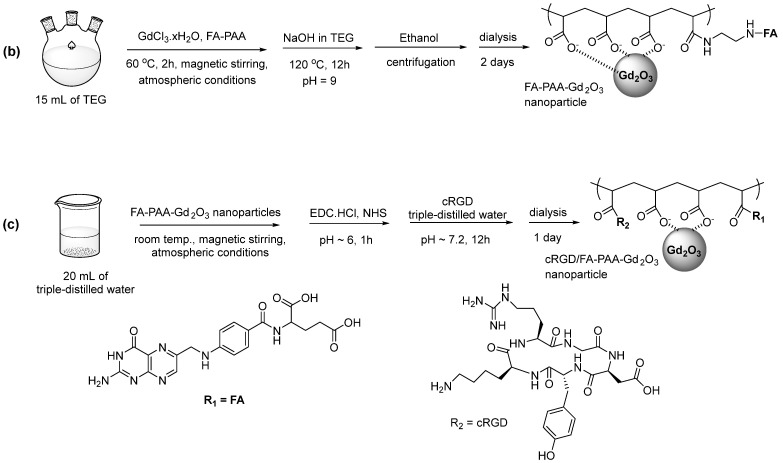
(**a**) Synthesis of FA-PA, (**b**) one-pot polyol synthesis of FA-PAA-Gd_2_O_3_ nanoparticles, and (**c**) synthesis of cRGD/FA-PAA-Gd_2_O_3_ nanoparticles.

FA-PAA was first prepared as described previously (Figure 1a) [40]. To obtain FA-NH_2_-Boc, 0.9 mmol FA was dissolved in DMSO (15 mL) in a 100-mL three-neck round-bottom flask at 60 °C under N_2_ flow with magnetic stirring. After the solution attained room temperature, 1.0 mmol DCC and 0.1 mmol DMAP were dissolved in the solution by magnetic stirring for 1 h. Next, 1.8 mmol EDA-Boc was dissolved in the solution by magnetic stirring for another 12 h. The resulting solution was slowly poured into cold ethyl acetate, and finally FA-EDA-Boc (yellow precipitate) was washed several times with ethyl acetate. Synthesis of FA-NH_2_-TFA was carried out by dissolving the yellow precipitate in 2 mL TFA in a 100-mL three-neck round-bottom flask with magnetic stirring for 3 h at room temperature. Chloroform was slowly poured into the solution until a yellow precipitate was obtained. Next, the clear solution was removed and precipitate was washed three times with ethyl acetate. The obtained FA-NH_2_-TFA was dried to powdered form using a rotary evaporator. To obtain FA-PAA, FA-NH_2_-TFA was dissolved in 5 mL DMSO containing 40 μL TEA with magnetic stirring. Separately, 1.5 mmol PAA was dissolved in DMSO (20 mL) under N_2_ flow at 60 °C in a 100-mL three-neck round-bottom flask with magnetic stirring. After the solution attained room temperature, 1.5 mmol DCC and 0.15 mmol DMAP were dissolved in the solution with continuous magnetic stirring for 1 h. Then, the above-prepared FA-NH_2_-TFA solution was slowly added to the PAA solution with continuous magnetic stirring for 12 h. The obtained solution was dialyzed against triple-distilled water for 24 h (MWCO = 1000 amu). The remaining solution inside the bag was filtered through Whatman filter paper (Sigma-Aldrich, USA) and evaporated using a rotary evaporator to collect FA-PAA (dark yellow solid).

FA-PAA-Gd_2_O_3_ nanoparticles were obtained using a one-pot polyol method (Figure 1b). Briefly, a mixture of 2.0 mmol GdCl_3_∙6H_2_O, 0.3 mmol of the above-synthesized FA-PAA, and 20 mL TEG was magnetically stirred in a three-neck round-bottom flask at 60 °C under atmospheric conditions to prepare a clear precursor solution. Next, NaOH (10 mmol) dissolved in 10 mL TEG was slowly poured into the precursor solution with magnetic stirring for 12 h at 120 °C until the pH reached ~9.0. Subsequently, the solution was cooled to room temperature and 400 mL ethanol was poured with magnetic stirring for ~30 min. FA-PAA-Gd_2_O_3_ nanoparticles were obtained by centrifugation (4000 rpm) and removing the supernatant. The nanoparticles were finally dispersed in ethanol, followed by centrifugation, and this step was repeated five times to remove TEG, free ions (Gd^3+^, Na^+^, and Cl^−^), and unreacted FA-PAA. Finally, the product solution was dialyzed against triple-distilled water (MWCO = 2000 amu) for two days to remove any remaining impurities from the FA-PAA-Gd_2_O_3_ nanoparticles.

### 2.3. Preparation of cRGD/FA-PAA-Gd_2_O_3_ Nanoparticles

Three quarters of the synthesized FA-PAA-Gd_2_O_3_ nanoparticles, 1.0 mmol EDC∙HCl, and 1.0 mmol NHS were added to 20 mL triple-distilled water at room temperature under atmospheric conditions (Figure 1c). The solution pH was maintained at 6.0 by adding 1.0 M HCl with magnetic stirring at room temperature for 1 h. The solution pH was then increased to 7.2 by adding 1.0 M NaOH, followed by adding 50 mg cRGD. The resulting solution was stirred magnetically for 12 h followed by dialysis against triple-distilled water (MWCO = 1000 amu) for one day to remove free cross-linking agents and unreacted cRGD. A portion of the COOH groups in PAA was conjugated with Gd_2_O_3_ nanoparticles via hard acid (i.e., Gd^3+^) and hard base (i.e., COO^−^) bonding, and a portion of the remainder was conjugated with NH_2_ groups of FA and cRGD via amide bonds.

### 2.4. Evaluation of Physicochemical Properties of the Nanoparticles

To measure the nanoparticle diameters, a high-resolution transmission electron microscope (HRTEM) (200 kV; FEI, Hillsboro, OR, USA; Titan G2 ChemiSTEM CS Probe) was used. The colloidal nanoparticles dispersed in aqueous media were dropped using a micropipette (2–20 μL, Eppendorf, Hamburg, Germany) onto a carbon film supported by a 200-mesh copper grid (Ted Pella Inc., Redding, CA, USA; Pelco No. 160) and air-dried at room temperature. Subsequently, the elements (C, N, O, and Gd) present in the nanoparticles were identified by an energy-dispersive X-ray spectroscope (EDS) (Bruker, Berlin, Germany; Quantax Nano) installed inside the HRTEM. To measure the Gd concentration in nanoparticle suspension, inductively coupled plasma–atomic emission spectroscopy (ICP-AES) (Thermo Jarrell Ash Co., Waltham, MA, USA; IRIS/AP) was used. The hydrodynamic diameters (a) and zeta potentials (**ζ**) of the nanoparticle colloids (0.01 mM Gd) were characterized using a dynamic light scattering (DLS) particle size analyzer (Malvern, Malvern, UK; Zetasizer Nano ZS). The nanoparticle structures in the powdered samples were characterized using an X-ray diffraction (XRD) machine (Philips, The Netherlands; X’PERT PRO MRD) with unfiltered CuKa (λ = 0.154184 nm) radiation; a scan range of 15–100° and a scanning step of 0.033° in 2θ were used. FT-IR absorption spectra (Mattson Instrument Inc., Madison, WI, USA; Galaxy 7020A) were taken using the powdered sample pellets with KBr to investigate PAA conjugation with nanoparticles, cRGD, and FA within 400–4000 cm^−1^. The surface-coating amount was quantified using a thermo-gravimetric analysis (TGA) instrument (TA Instrument, New Castle, DE, USA; SDT-Q600) between room temperature and 900 °C under air flow. The average amounts (in wt.%) of surface-coating ligands (FA-PAA and cRGD/FA-PAA) were obtained from the mass drops in TGA curves after considering water and air desorption between room temperature and ~105 °C. The amount of nanoparticles was obtained from the remaining mass followed by XRD analysis. Elemental analysis (EA) (ThermoFisher, Waltham, MA, USA; Flash 2000) was carried out to measure the composition (C/H/O/N) and amount of surface-coating ligands (in wt.%) using powdered samples.

### 2.5. In Vitro Cellular Cytotoxicity Assay

Normal mouse hepatocytes (NCTC1469) and human malignant glioma (U87MG) cell lines were cultured in DMEM and RPMI-1640 media, respectively. Cells (5 × 10^4^) were seeded into 24-well plates (500 μL cells/well) and incubated for 24 h in 5% CO_2_ at 37 °C. The concentrated nanoparticle suspension was diluted with sterile PBS solution to prepare five test concentrations. Subsequently, 2 μL aliquots were added to the cells to obtain 10, 50, 100, 200, and 500 μM Gd concentrations, followed by 48 h incubation. Next, 200 μL CellTiter-Glo reagent was added for cell lysis and the reaction was incubated on an orbital shaker for 30 min. The cellular cytotoxicity of the nanoparticle suspension samples was measured using a CellTiter-Glo Luminescent Cell Viability Assay (Promega, Madison, WI, USA) according to the manufacturer’s instructions. Intracellular adenosine triphosphate was quantified using a Victor 3 luminometer (Perkin Elmer, Waltham, MA, USA) in the 300–700 nm wavelength range. Cell viability was measured in triplicate to obtain average values, which were normalized to those of the untreated control cells (0.0 mM Gd).

### 2.6. Water Proton Spin Relaxivity and Map Image Measurements

The concentrated nanoparticle suspension was diluted with triple-distilled water to prepare various concentrations (1, 0.5, 0.25, 0.125, and 0.0625 mM Gd), which were subject to analysis of the longitudinal (T_1_) and transverse (T_2_) water proton spin relaxation times and longitudinal (R_1_) and transverse (R_2_) map images using a 3.0 T MRI scanner (Siemens, Munich, Germany; Magnetom Trio Tim). Next, inverse relaxation times (1/T_1_ and 1/T_2_) were plotted as a function of Gd concentration to estimate the r_1_ and r_2_ values from the corresponding slopes. An inversion recovery method was used to measure the T_1_ relaxation times by recording MR images at 35 different inversion times (TI) in the range of 50–1750 ms. The T_1_ values were estimated from nonlinear least-square fits to the mean signal intensities at various TI values. To measure T_2_ relaxation times, the Carr–Purcell–Meiboom–Gill pulse sequence was used for multiple spin-echo measurements. The MR images were obtained at 16 different echo time (TE) values in the range of 10–190 ms. The T_2_ values were estimated from the nonlinear least-square fits to the mean signal intensities of the multiple spin-echo measurements at various TE values. The following parameters were used for measurements: MR field (H) = 3.0 T, temperature (T) = 22 °C, repetition time (TR) = 2000 ms, field of view (FOV) = 16 cm, FOV phase = 0.5, slice thickness = 5 mm, number of acquisitions (NEX) = 1, pixel spacing = 0.625 mm, pixel band width = 122.10 Hz, and matrix size = 256 × 128.

### 2.7. Preparation of Murine Tumor Model

U87MG tumor cells were cultured in RPMI-1640 containing 10% (v/v) fetal bovine serum and 1% (v/v) penicillin streptomycin for 24 h in 5% CO_2_ at 37°. Six 5-week-old male BALB/c nude mice (~20 g) were injected subcutaneously with U87MG tumor cells (5 × 10^6^ cells/100 μL of PBS) in the left rump tissue, and MRI experiments were carried out after three weeks.

### 2.8. In Vivo T_1_ MR Image Measurements

Mice were anesthetized using 1.5% isoflurane in oxygen. Measurements were taken before and after injecting the two forms of aqueous nanoparticle suspensions (approximately 0.1 mmol Gd/kg) into the tail veins of mice (*N* = 3 each group). A warm water blanket was used to maintain the body temperature at 37 °C during measurements. The slight breathing movements of mice, even under anesthesia, were fixed using a small animal sleeve. In addition, the mice were wrapped with a band around their abdomens to minimize abdominal movements. After the measurements, the mice were revived from anesthesia and placed in cages with free access to food and water. Radio frequency-spoiled T_1_-weighted gradient-recalled echo (GRE) sequences were used for obtaining images. The experimental parameters were as follows: H = 3.0 T, T = 37 °C, TE = 7 ms, TR = 850 ms, pixel band width = 15.63 Hz, frequency = 256 Hz, phase = 256, NEX = 3, FOV = 60 mm, FOV phase = 1, slice thickness = 1.0 mm, number of slices = 24, and spacing gap = 1.1 mm. The signal-to noise ratio (SNR) was defined as the ratio of mean signal intensity of the anatomical region of interest (ROI) to that of the background noise. The T_1_-contrast ROI was defined as SNR (t)/SNR (0), with t the time after injection and 0 the time before injection.

## 3. Results

### 3.1. Physicochemical Properties of FA-PAA-Gd_2_O_3_ and cRGD/FA-PAA-Gd_2_O_3_ Nanoparticles

**Figure 2:**
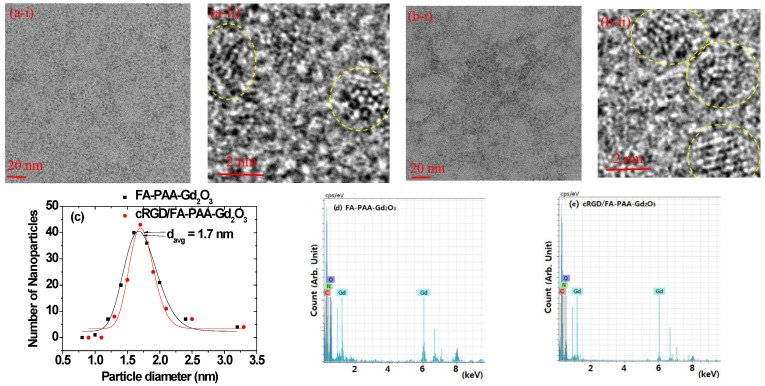
TEM images of (**a-i**,**a-ii**) FA-PAA-Gd_2_O_3_ and (**b-i**,**b-ii**) cRGD/FA-PAA-Gd_2_O_3_ nanoparticles at 20 and 2 nm scales. The yellow circles indicate individual nanoparticles. (**c**) Log-normal function fit of the observed particle diameter distributions to estimate d_avg_ values. EDS spectra of (**d**) FA-PAA-Gd_2_O_3_ and (**e**) cRGD/FA-PAA-Gd_2_O_3_ nanoparticles to confirm presence of C, O, N, and Gd in the nanoparticles.

The FA-PAA-Gd_2_O_3_ (Figure 2(a-i,a-ii)) and cRGD/FA-PAA-Gd_2_O_3_ nanoparticles (Figure 2(b-i,b-ii)) were nearly monodispersed and ultrasmall, with diameters ranging from 1.5–3.0 nm. The average particle diameters (d_avg_) of FA-PAA-Gd_2_O_3_ and cRGD/FA-PAA-Gd_2_O_3_ were 1.7 nm, as estimated from log-normal function fits to the observed particle diameter distributions (Figure 2c). The EDS spectra confirmed the presence of Gd, C, N, and O in the nanoparticles (Figure 2d,e). The observed values are listed in Table 1.

**Figure 3:**
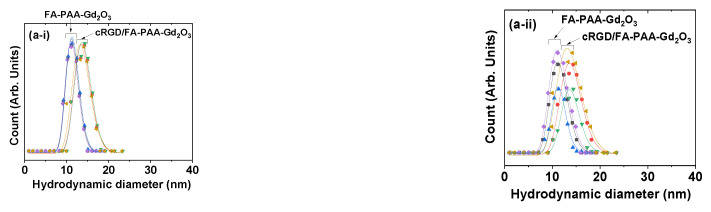

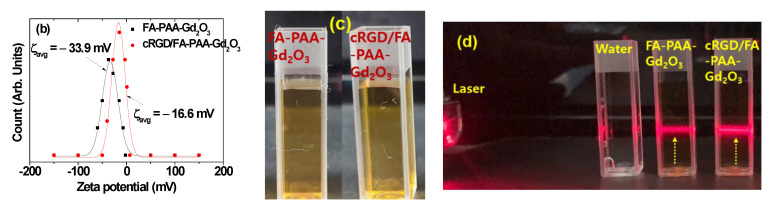
DLS patterns of the FA-PAA-Gd_2_O_3_ and cRGD/FA-PAA-Gd_2_O_3_ nanoparticles in (**a-i**) aqueous media and (**a-ii**) physiological solution (0.9 NaCl wt.% in water); the a_avg_ values as estimated using log-normal function fits to the observed DLS patterns. DLS patterns were measured three times; (■, ▲, ♦) for FA-PAA-Gd_2_O_3_ nanoparticles and (●, ▼, ◄) for cRGD/FA-PAA-Gd_2_O_3_ nanoparticles. (**b**) Zeta potentials of the FA-PAA-Gd_2_O_3_ and cRGD/FA-PAA-Gd_2_O_3_ nanoparticles in aqueous media. (**c**) FA-PAA-Gd_2_O_3_ and cRGD/FA-PAA-Gd_2_O_3_ nanoparticles in aqueous media, exhibiting excellent colloidal stability (no precipitation for >1 year after synthesis). (**d**) Tyndall effect, confirming the colloidal dispersion of the FA-PAA-Gd_2_O_3_ and cRGD/FA-PAA-Gd_2_O_3_ nanoparticles in aqueous media; no laser light scattering is observed in triple-distilled water. Arrows indicate laser light scattering by the nanoparticle colloids.

The hydrodynamic diameters (a_avg_) of FA-PAA-Gd_2_O_3_ and cRGD/FA-PAA-Gd_2_O_3_ nanoparticles dispersed in aqueous media and physiological solution (0.9 NaCl wt.% in water) were measured to be 11.4 and 13.8 nm, respectively, by their DLS patterns (Figure 3(a-i,a-ii)) using log-normal function fits to the observed hydrodynamic diameter distributions (Table 1). DLS patterns were measured three times. Similar hydrodynamic diameters were observed for both samples at all times, indicating the presence of stable colloids in aqueous and physiological solutions. PAA contains a large number of hydrophilic COOH groups; therefore, the FA-PAA-Gd_2_O_3_ and cRGD/FA-PAA-Gd_2_O_3_ nanoparticles can attract a large number of water molecules, which explains the observed large a_avg_ values and excellent colloidal stability. Moreover, the cRGD/FA-PAA-Gd_2_O_3_ nanoparticles had a higher a_avg_ value than the FA-PAA-Gd_2_O_3_ nanoparticles due to the additional cRGDs in their surface-coating layers. Additionally, the lesser number of free COO^−^ groups in the cRGD/FA-PAA-Gd_2_O_3_ nanoparticles resulted in their lower zeta potential (ζ; −16.6 mV) than that (−33.9 mV) of the FA-PAA-Gd_2_O_3_ nanoparticles (Figure 3b and Table 1). As shown in Figure 3c, the aqueous nanoparticle suspensions exhibited excellent colloidal stability (i.e., no precipitation after synthesis for >1 year). The dispersion of nanoparticle colloids in aqueous media was confirmed by the Tyndall effect (Figure 3d); laser light scattering was observed only in nanoparticle suspension samples (two cuvettes on the right), unlike in triple-distilled water (left cuvette).

### 3.2. Crystal Structures of the Nanoparticles

**Figure 4:**
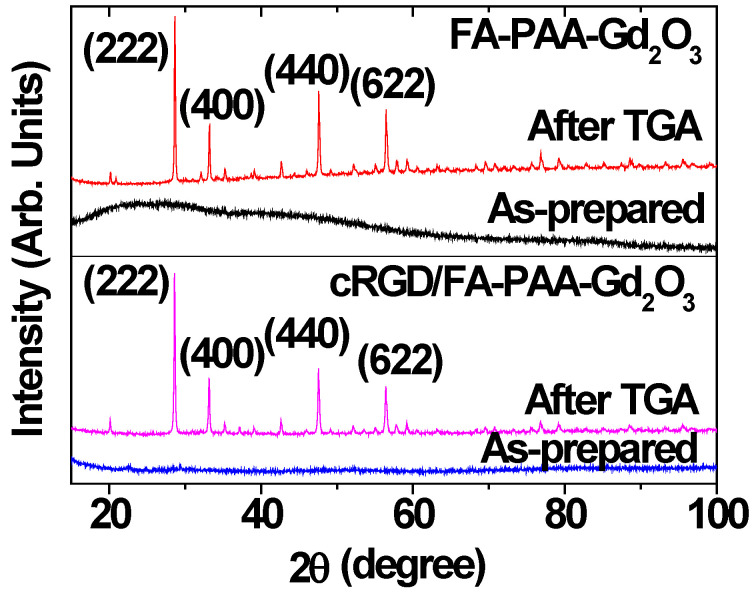
XRD patterns of the FA-PAA-Gd_2_O_3_ and cRGD/FA-PAA-Gd_2_O_3_ nanoparticles before (i.e., as-prepared) and after TGA. The representative assignments on XRD peaks after TGA are the (hkl) Miller indices of cubic Gd_2_O_3_. All observed peaks could be assigned with the (hkl) Miller indices of cubic Gd_2_O_3_.

The XRD patterns of FA-PAA-Gd_2_O_3_ and cRGD/FA-PAA-Gd_2_O_3_ nanoparticles were recorded before and after TGA (Figure 4). Prior to TGA, the nanoparticles were not fully crystallized owing to their ultrasmall particle size, resulting in broad amorphous XRD patterns [41]. However, crystal growth during TGA up to 900 °C led to sharp peaks of body-centered cubic (bcc) Gd_2_O_3_ [42]. Moreover, the powdered samples subjected to TGA showed a lattice constant of 10.814 Å, which is consistent with the reported value (10.813 Å) [42].

### 3.3. Surface Coatings

**Figure 5:**
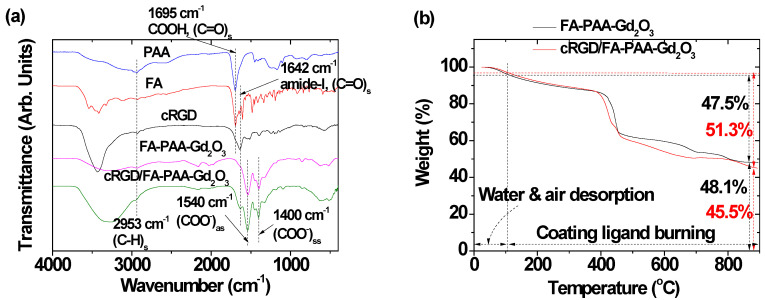
(**a**) FT-IR absorption spectra of PAA, FA, cRGD, FA-PAA-Gd_2_O_3_, and cRGD/FA-PAA-Gd_2_O_3_ nanoparticles. (**b**) TGA curves of the FA-PAA-Gd_2_O_3_ and cRGD/FA-PAA-Gd_2_O_3_ nanoparticles.

The surface coating of ultrasmall Gd_2_O_3_ nanoparticles with FA-PAA and cRGD/FA-PAA was supported by FT-IR absorption spectra (Figure 5a). The C=O stretching vibration of the COOH groups of PAA at 1695 cm^−1^ exhibited red-shift and split into COO^−^ antisymmetric and symmetric stretching vibrations at 1540 and 1400 cm^−1^, respectively [43], confirming the successful coating of PAA on the ultrasmall Gd_2_O_3_ nanoparticle surface. The red-shift and split resulted from the hard acid–base bonding between the COO^−^ (hard base) of PAA and Gd^3+^ (hard acid) of the Gd_2_O_3_ nanoparticles [44]. Additionally, the C–H stretching vibrations of PAA, FA, and cRGDs at ~2953 cm^−1^ were observed in the FT-IR absorption spectra of FA-PAA-Gd_2_O_3_ and cRGD/FA-PAA-Gd_2_O_3_ nanoparticles, supporting the presence of these ligands in the nanoparticles. Amide-I C=O stretching vibration of FA and cRGD (at 1642 cm^−1^) [45,46] was observed as well, confirming the successful conjugation of NH_2_ groups of FA and cRGD with the COOH groups of PAA.

The surface-coating amount (P; in wt.%) was obtained by TGA. As shown in Figure 5b, the *p* values were 47.5 and 51.3% for FA-PAA-Gd_2_O_3_ and cRGD/FA-PAA-Gd_2_O_3_ nanoparticles (Table 1), respectively, as determined by the mass loss after taking into account water and air desorption between room temperature and ~105 °C. The remaining mass was ascribed to Gd_2_O_3_ nanoparticles (Figure 5b and Table 1). The cRGD/FA-PAA-Gd_2_O_3_ nanoparticles had a higher *p* than that of the FA-PAA-Gd_2_O_3_ nanoparticles due to additional cRGDs in their structure. Based on the EA, *p* values were 52.4 and 56.8% for FA-PAA-Gd_2_O_3_ and cRGD/FA-PAA-Gd_2_O_3_ nanoparticles, respectively, as determined by adding the C/H/O/N atomic wt.%, i.e., 22.57/3.48/25.01/1.32 and 24.44/3.75/25.86/2.76, respectively. These values were slightly higher than those estimated by TGA because the water and air content in the samples could not be eliminated from the measured wt.% in EA. The estimated wt.% difference (i.e., 3.8% by TGA and 4.4% by EA, for an average of 4.1%) between cRGD/FA-PAA-Gd_2_O_3_ and FA-PAA-Gd_2_O_3_ nanoparticles represented the wt.% of cRGD. Assuming that the PAA/FA molar ratio of 1.5/0.9 used in FA-PAA synthesis was maintained in the nanoparticle samples, the wt.% of cRGD/FA/PAA was estimated as 4.1/6.8/45.9. Based on the bulk density of Gd_2_O_3_ (7.407 g/cm^3^) [47], *p* values estimated from TGA and EA, average mass of FA-PAA (2064 g) and cRGD/FA-PAA (2225 g) obtained using the above-estimated ligand wt.% ratio, and d_avg_ value determined by HRTEM imaging, the grafting density (σ, i.e., the average number of FA-PAA and cRGD/FA-PAA coating a Gd_2_O_3_ nanoparticle unit surface area) [48] was found to be 0.6–0.7 nm^−2^. By multiplying σ with the Gd_2_O_3_ nanoparticle surface area (πd_avg_^2^), the average number (N_NP_) of FA-PAA and cRGD/FA-PAA coating each Gd_2_O_3_ nanoparticle was found to be 6–7. The surface-coating results are listed in Table 1.

### 3.4. r_1_, r_2_ Values and R_1_, R_2_ Map Images

**Figure 6:**
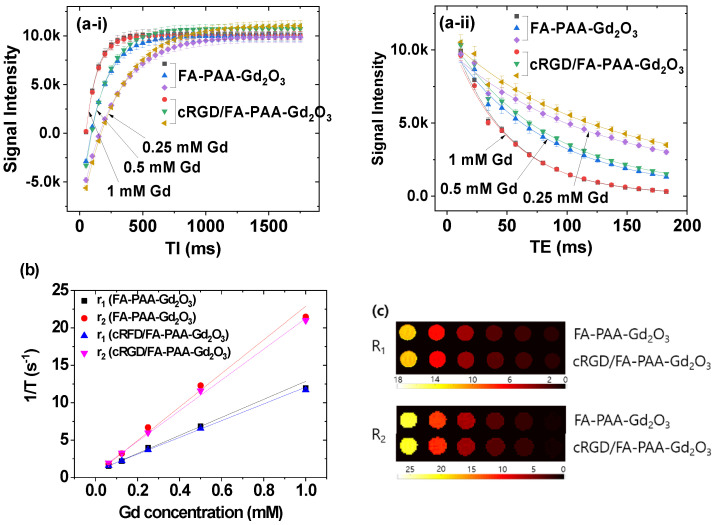
Nonlinear least-square fits to (**a-i**) the measured mean signal intensities at various TI values to obtain T_1_ relaxation times and (**a-ii**) the mean signal intensities of multiple spin-echo measurements at various TE values to obtain T_2_ relaxation times. (**b**) Plots of 1/T_1_ and 1/T_2_ inverse relaxation times as a function of Gd concentration for FA-PAA-Gd_2_O_3_ and cRGD/FA-PAA-Gd_2_O_3_ nanoparticles in aqueous media at H = 3.0 T and 22 °C. The slopes correspond to r_1_ and r_2_ values, respectively. (**c**) R_1_ and R_2_ map images showing dose-dependent contrast enhancements.

To investigate the potential of the synthesized FA-PAA-Gd_2_O_3_ and cRGD/FA-PAA-Gd_2_O_3_ nanoparticles as T_1_ MRI contrast agents, T_1_ and T_2_ relaxation times were measured at various Gd concentrations at 3.0 T MR field and 22 °C. For 0.25, 0.5, and 1.0 mM Gd, non-linear curve fits to obtain the T_1_ and T_2_ relaxation times are provided in Figure 6(a-i,a-ii), respectively. Subsequently, 1/T_1_ and 1/T_2_ inverse relaxation times were plotted as a function of Gd concentration to obtain r_1_ and r_2_ values from the corresponding slopes (Figure 6b and Table 2). As shown in Table 2, the estimated r_1_ values were approximately four times higher than those of commercial Gd-chelates [49]. In addition, the synthesized nanoparticles exhibited dose-dependent contrast changes in R_1_ and R_2_ map images (Figure 6c). Considering that the r_2_/r_1_ ratios were close to 1, these results indicate that the synthesized nanoparticles could act as high-performance T_1_ MRI contrast agents.

The r_2_/r_1_ ratio is greater than 1 because longitudinal relaxation accompanies transverse relaxation, whereas the reverse is not feasible. Therefore, r_2_/r_1_ ratios close to 1 and as large as possible are ideal for T_1_ and T_2_ MRI contrast agents, respectively. Therefore, Gd-chelates and iron oxide nanoparticles are suitable for use as T_1_ and T_2_ MRI contrast agents, respectively. Similarly, FA-PAA-Gd_2_O_3_ and cRGD/FA-PAA-Gd_2_O_3_ nanoparticles are potential T_1_ MRI contrast agents, as their r_2_/r_1_ ratios are close to 1.

### 3.5. Cellular Toxicity of the Nanoparticles

**Figure 7:**
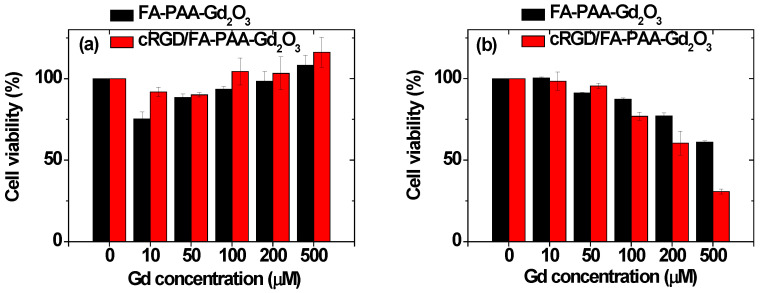
Plots showing viability of (**a**) NCTC1469 (normal) and (**b**) U87MG (tumor) cells after 48 h of incubation with FA-PAA-Gd_2_O_3_ and cRGD/FA-PAA-Gd_2_O_3_ nanoparticles.

The toxicity of FA-PAA-Gd_2_O_3_ and cRGD/FA-PAA-Gd_2_O_3_ nanoparticles was investigated by measuring the viability of NCTC1469 normal and U87MG tumor cells. As shown in Figure 7a, NCTC1469 cells exhibited good viability when treated with up to 500 μM Gd in both nanoparticle samples. However, the viability of U87MG cells decreased with increasing Gd concentration (Figure 7b). The toxicity observed in U87MG cells was attributed to the overexpressed receptors and integrins in tumor cells compared with those in normal cells and the resultant targeting effect of nanoparticles [31,32,33,34,35,36,37,38,39]. In addition, at high Gd concentrations, increased cellular toxicity of the cRGD/FA-PAA-Gd_2_O_3_ nanoparticles compared to that of the FA-PAA-Gd_2_O_3_ nanoparticles was attributed to multiple targeting by cRGD and FA in the cRGD/FA-PAA-Gd_2_O_3_ nanoparticles.

Recently, enhanced cytosolic concentration of reactive oxygen species (ROS) and autophagic vesicles has been reported as a result of internalized gadolinium oxide nanoparticles in human umbilical vein endothelial and breast cancer cells (MCF-7) [50,51]. Consequently, potential mitochondrial membrane collapse, cell viability reduction, and cell death via necrosis and apoptosis were observed. In addition, growing evidence supports nanoparticle-induced ROS and subsequent ROS-mediated cellular apoptosis and necrosis for various nanoparticle systems [52,53,54]. Similar cytotoxic effects probably decreased U87MG cell viability with increasing Gd concentration in the present study. However, detailed studies are needed to unfold the mechanisms underlying FA-PAA-Gd_2_O_3_ and cRGD/FA-PAA-Gd_2_O_3_ nanoparticle-mediated cytotoxicity in U87MG tumor cells.

### 3.6. In Vivo T_1_ MRI

**Figure 8:**
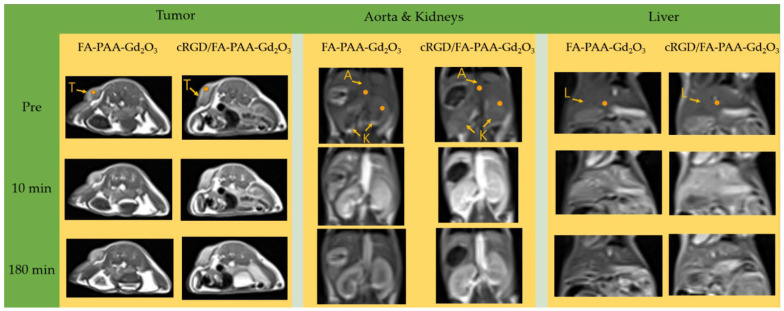
T_1_ MR images at a 3.0 T MR field as a function of time before (labeled as “Pre”) and after intravenous injection of aqueous FA-PAA-Gd_2_O_3_ and cRGD/FA-PAA-Gd_2_O_3_ nanoparticle suspension samples into mice tails. “T” denotes the tumor, “A” denotes the aorta, “K” denotes the kidneys, and “L” denotes the liver. The regions of interest (ROIs) are labeled with small dots.

**Figure 9:**
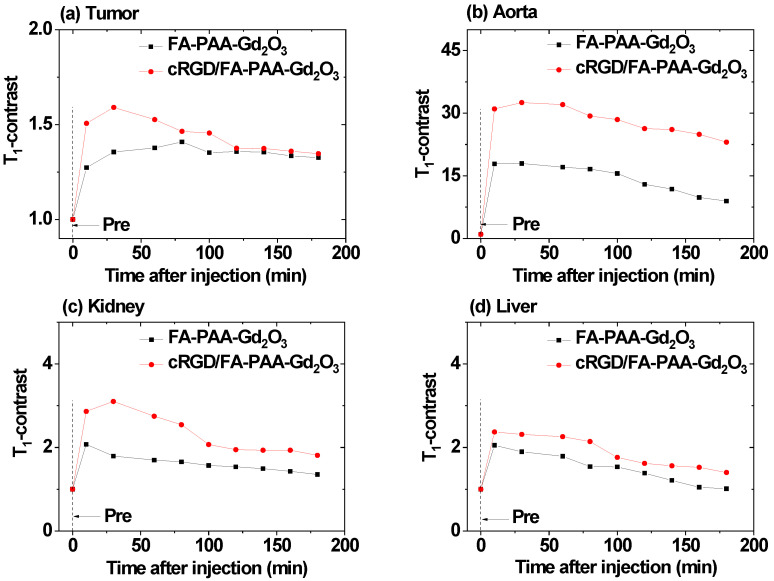
Plots and comparison of T_1_-contrast ROIs between the FA-PAA-Gd_2_O_3_ and cRGD/FA-PAA-Gd_2_O_3_ nanoparticles in (**a**) the tumor, (**b**) aorta, (**c**) kidneys, and (**d**) liver as a function of time before and after intravenous injection of the nanoparticle suspension samples into mice tails. T_1_-contrast ROI = SNR (t)/SNR (0).

T_1_ MR images of the tumor and organs including the liver, kidneys, and aorta were measured before and after intravenous injection (up to 3 h) of the aqueous nanoparticle suspension samples into mice tails (Figure 8). Positive contrasts were observed in the tumor and all organs after injection, confirming that the nanoparticle samples acted as T_1_ MRI contrast agents. To study the contrast changes with time, the T_1_-contrast of the ROI were plotted as a function of time (Figure 9a–d), and they increased to reach maxima within an hour after injection, followed by a decrease thereafter. Notably, the T_1_-contrast ROIs were the highest in the aorta, followed by the kidneys for both FA-PAA-Gd_2_O_3_ and cRGD/FA-PAA-Gd_2_O_3_ nanoparticles, indicating their prolonged blood circulation and delayed renal excretion. In addition, the T_1_-contrast ROIs of the cRGD/FA-PAA-Gd_2_O_3_ nanoparticles were higher than those of the FA-PAA-Gd_2_O_3_ nanoparticles for tumors and all organs, confirming that cRGD enhanced tumor imaging and prolonged the blood circulation duration.

## 4. Discussion

In the present study, mono (i.e., FA) and multiple (i.e., cRGD and FA) tumor-targeting ligand-coated ultrasmall Gd_2_O_3_ nanoparticles were synthesized. FA-PAA-Gd_2_O_3_ and cRGD/FA-PAA-Gd_2_O_3_ nanoparticles were nearly monodispersed with an average particle diameter of 1.7 nm. The hydrodynamic diameters were 11.4 and 13.8 nm and zeta potentials were −33.9 and −16.6 mV for FA-PAA-Gd_2_O_3_ and cRGD/FA-PAA-Gd_2_O_3_ nanoparticles, respectively. Their colloidal stability was excellent, as the nanoparticles did not precipitate for more than one year after synthesis. Both nanoparticle samples exhibited approximately four times higher r_1_ values compared with those of the commercial molecular chelates [49], confirming their potential as high-performance T_1_ MRI contrast agents.

Both forms of the nanoparticles did not show any toxicity in NCTC1469 cells up to 500 μM Gd concentration. However, increased toxicity was observed in U87MG cells with increasing Gd concentration (Figure 7b). This was attributed to the tumor-targeting effect of the nanoparticles. In addition, the toxicity of the cRGD/FA-PAA-Gd_2_O_3_ nanoparticles was slightly higher than that of the FA-PAA-Gd_2_O_3_ nanoparticles because of multiple tumor targeting by cRGD and FA in the cRGD/FA-PAA-Gd_2_O_3_ nanoparticles.

**Figure 10:**
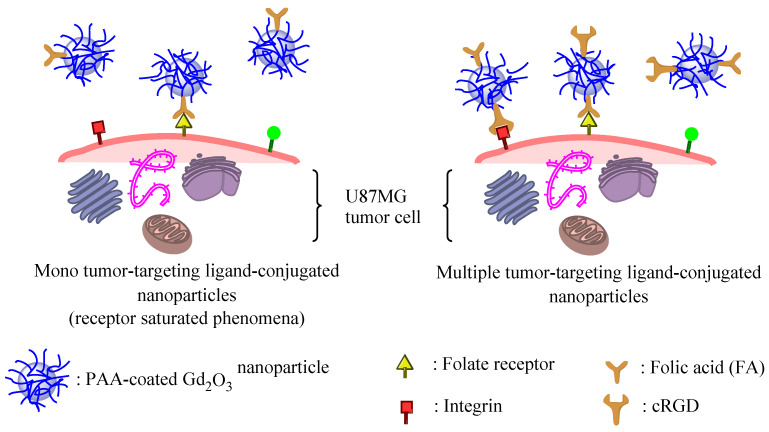
Schematic diagram showing enhanced tumor imaging by the cRGD/FA-PAA-Gd_2_O_3_ nanoparticles (**right**) compared with the FA-PAA-Gd_2_O_3_ nanoparticles (**left**).

Additionally, the T_1_-contrast ROIs of cRGD/FA-PAA-Gd_2_O_3_ nanoparticles in the tumor were higher than those of the FA-PAA-Gd_2_O_3_ nanoparticles (Figure 9a). This demonstrates the superiority of the multiple-targeting over the mono-targeting approach for tumor imaging. As shown in Figure 10, a mono tumor-targeting ligand (i.e., FA in FA-PAA-Gd_2_O_3_ nanoparticles) only targets the folate receptors overexpressed on U87MG tumor cells (termed receptor saturation phenomena; left figure in Figure 10), whereas multiple tumor-targeting ligands (i.e., FA and cRGD in cRGD/FA-PAA-Gd_2_O_3_ nanoparticles) target folate receptors as well as integrins (right figure in Figure 10), leading to improved tumor imaging as well as enhanced tumor cytotoxicity.

Both FA-PAA-Gd_2_O_3_ and cRGD/FA-PAA-Gd_2_O_3_ nanoparticles exhibited the highest positive contrasts in the aorta among the organs analyzed, which included the liver, kidneys, and tumors (Figure 9a–d), showing their prolonged circulation in the blood. Nanoparticles that can circulate in the blood for prolonged durations should have hydrodynamic diameters small enough to minimize opsonization [14,15] and evade RES uptake, and large enough (>10 nm) to delay renal excretion [14,16]. The hydrodynamic diameters of the nanoparticles synthesized in this study ranged from 11 to 14 nm, thereby satisfying these conditions.

As shown in Figure 9b–d, cRGD/FA-PAA-Gd_2_O_3_ nanoparticles exhibited higher positive contrasts in all organs, especially in the aorta, than the FA-PAA-Gd_2_O_3_ nanoparticles. This was likely due to the blood circulation-enhancing effect of the cRGD present in the cRGD/FA-PAA-Gd_2_O_3_ nanoparticles. As cRGD binds to integrins expressed on blood cells (termed cell hitchhiking) [14,55], the nanoparticles can circulate for a longer duration in the blood and provide a better contrast.

It is known that nanoparticles with ultrasmall particle and hydrodynamic diameters (d < 3 nm and a < 5 nm) are excretable via the renal system [56,57]. The synthesized nanoparticles (d_avg_ = 1.7 nm) in the present study exhibited a_avg_ = 11.4 nm for FA-PAA-Gd_2_O_3_ nanoparticles and 13.8 nm for cRGD/FA-PAA-Gd_2_O_3_ nanoparticles. Therefore, a portion of the nanoparticles could be slowly excreted through the renal system, as can be noticed from the gradual decrease in SNR with time in the kidneys (Figure 9c). However, detailed studies are needed to clarify the excretion pathway of the nanoparticles. Ultrasmall nanoparticles exhibited no or negligible contrast enhancements in healthy normal brain MRI [58], supporting that they cannot pass the blood–brain barrier (BBB) for the normal brain; however, they can pass the BBB for brain tumors, possibly through damage to the BBB, as observed in brain tumor MRI with D-glucuronic acid-coated ultrasmall Gd_2_O_3_ nanoparticles [59]. For other organ tumors, tumor-targeting ligand-conjugated Gd_2_O_3_ nanoparticles have been successfully applied to tumor imaging via various imaging modalities [5]. The toxicity of Gd_2_O_3_ nanoparticles is of great concern owing to the release of Gd^3+^ ions [60,61,62]. For commercial molecular Gd^3+^-chelates, it is known that if free Gd^3+^ ions are liberated in the body, this can promote nephrogenic systemic fibrosis, which is a rare disease that can lead to hardening or thickening of the skin and deposits [63]; therefore, Gd_2_O_3_ nanoparticles should be completely excreted through the renal system after injection.

## 5. Conclusions

Hydrophilic and biocompatible PAA-coated ultrasmall Gd_2_O_3_ nanoparticles (d_avg_ = 1.7 nm) were successfully conjugated with the tumor-targeting ligands FA and/or cRGD. The FA-PAA-Gd_2_O_3_ and cRGD/FA-PAA-Gd_2_O_3_ nanoparticles exhibited excellent colloidal stability (no precipitation for >1 year after synthesis). They were successfully applied for tumor imaging in U87MG tumor-bearing mice via T_1_ MRI. The salient outcomes of our study can be summarized as follows:

(1) Both nanoparticles displayed r_1_ values approximately four times higher (12.0 and 11.2 s^−1^ mM^−1^ for FA-PAA-Gd_2_O_3_ and cRGD/FA-PAA-Gd_2_O_3_ nanoparticles, respectively) than those of commercially available Gd-chelates.
(2) The cRGD/FA-PAA-Gd_2_O_3_ nanoparticles exhibited higher contrasts at the tumor site than the FA-PAA-Gd_2_O_3_ nanoparticles owing to their multiple tumor-targeting effects.
(3) Both nanoparticles exhibited the highest contrast in the aorta among the various organs analyzed, because of prolonged blood circulation. This is due to their ideal hydrodynamic diameters (11.4 and 13.8 nm for FA-PAA-Gd_2_O_3_ and cRGD/FA-PAA-Gd_2_O_3_ nanoparticles, respectively), which are small enough to minimize opsonization and RES uptake and large enough to delay renal excretion.
(4) The cRGD/FA-PAA-Gd_2_O_3_ nanoparticles displayed higher contrasts in all organs, especially the aorta, compared with the FA-PAA-Gd_2_O_3_ nanoparticles, because of the blood cell hitchhiking phenomenon of cRGD in the cRGD/FA-PAA-Gd_2_O_3_ nanoparticles, which prolonged their circulation in the blood.

## Figures and Tables

**Table 1:** Physicochemical properties of FA-PAA-Gd_2_O_3_ and cRGD/FA-PAA-Gd_2_O_3_ nanoparticles.

Nanoparticle Sample	d_avg_ (nm)	a_avg_ (nm)	ζ (mV)	Surface Coating							
				P ^a^		Gd_2_O_3_ Nanoparticle		σ ^b^		N_NP_^c^	
				(wt.%)		(wt.%)		(1/nm^2^)			
				TGA	EA	TGA	EA	TGA	EA	TGA	EA
FA-PAA-Gd_2_O_3_	1.7	11.4	−33.9	47.5	52.4	48.1	47.6	0.6	0.7	5.5	6.1
cRGD/FA-PAA-Gd_2_O_3_	1.7	13.8	−16.6	51.3	56.8	45.5	43.2	0.6	0.7	5.8	6.8

^a^ Average ligand surface-coating amount in wt.%. ^b^ Grafting density, i.e., average number of ligands (FA-PAA or cRGD/FA-PAA) coating a unit surface area of a nanoparticle. ^c^ Average number of ligands coating a nanoparticle.

**Table 2:** r_1_ and r_2_ values of FA-PAA-Gd_2_O_3_ and cRGD/FA-PAA-Gd_2_O_3_ nanoparticles.

Chemical	Water Proton Spin Relaxivities in Aqueous Media at 3.0 T (s^−1^ mM^−1^)			Ref.
	r_1_	r_2_	r_2_/r_1_	
FA-PAA-Gd_2_O_3_	12.0	22.4	1.9	This work
cRGD/FA-PAA-Gd_2_O_3_	11.2	20.6	1.8	This work
Gd-DTPA	3.1	3.7	1.2	[49]
Gd-DOTA	2.8	3.3	1.2	[49]

## Data Availability

The data presented in this study are available on request from the corresponding authors.
